# Developing Sustainable Food Systems in Europe: National Policies and Stakeholder Perspectives in a Four-Country Analysis

**DOI:** 10.3390/ijerph18147701

**Published:** 2021-07-20

**Authors:** Alina Zaharia, Maria-Claudia Diaconeasa, Natalia Maehle, Gergely Szolnoki, Roberta Capitello

**Affiliations:** 1 Department of Agrifood and Environmental Economics, The Bucharest University of Economic Studies, 010371 Bucharest, Romania; alina.zaharia@eam.ase.ro (A.Z.); maria.diaconeasa@eam.ase.ro (M.-C.D.); 2 Mohn Centre for Innovation and Regional Development, Western Norway University of Applied Sciences, 5063 Bergen, Norway; natalia.mehle@hvl.no; 3 Department of Wine and Beverage Business Research, Geisenheim University, 65366 Geisenheim, Germany; gergely.szolnoki@hs-gm.de; 4 Department of Business Administration, University of Verona, 37129 Verona, Italy

**Keywords:** sustainable food, public policy, food policy, national policy documents, stakeholder interviews, critical frame analysis, multi-country analysis, food system

## Abstract

To address climate change, health, and food-related challenges at the international and regional level, policy makers and researchers are starting to acknowledge the importance of building and developing sustainable food systems (SFSs). This study aims to discuss the drivers of, barriers to, and policy recommendations for developing sustainable food systems in four European countries (Germany, Italy, Norway, and Romania). We used critical frame analysis to investigate national policy documents on sustainable food systems and conducted in-depth interviews with various national stakeholders representing policy makers, agrifood businesses, and civil society. The novelty of this research lies in comparing national policy approaches and stakeholders’ opinions on SFS development in a multi-country analysis. These European countries have different conditions in terms of geography, socioeconomic situation, environmental performance, and sustainability orientation. Several cross-cultural differences and gaps in the existing national policies for sustainable food systems were identified, and solutions that help overcome these issues have been suggested. The first step in developing SFS should focus on interdisciplinary and trans-sectorial policy integration combined with increasing stakeholder collaboration across all sectors of the economy. We also recommend more active involvement of consumers in the food system, developing information-sharing networks, and increasing collaborations within the food supply chains.

## 1. Introduction

The past century has seen a rapid increase in global challenges, both environmental and socioeconomic. This has resulted in the emergence of sustainable development rhetoric emphasising the need for systemic changes in the relationship between nature and humanity. Since 1987, when the Brundtland report institutionalised a sustainable development concept [1], various actors have combined their efforts to develop sustainable policies in different sectors, including agriculture and the food industry. International sustainability efforts were officially initiated at the 1992 Earth Summit in Rio de Janeiro and were recently globally extended through the 2030 Agenda for Sustainable Development and the 17 Sustainable Development Goals (SDGs) [2].

In this context, the European Union (EU) adopted several policies to increase sustainability in the food system—for example, promoting a circular economy, increasing resource efficiency, introducing sustainability ‘from farm to fork’, and ecosystem preservation and restoration [3]. Despite this, the European Commission [4] finds that its member states are still performing poorly on several of the SDGs, especially SDG 12 ‘Responsible consumption and production’ and SDG 14 ‘Life below water’. The EU governance structures seem to be ill-adapted to the systemic nature of food-related challenges, stressing the need for coherent policies stimulating more sustainable food practices [5,6].

While recognising the actions taken so far, the recent literature emphasises the gaps in European food policies. For example, several studies call for vertical and horizontal policy integration, improving coordination between the involved actors and increasing feedback loops within the multi-level governance [7,8]. It is also necessary to understand different issues related to building a holistic food system (e.g., introducing sustainable food standards and metrics), while at the same time taking into consideration regional differences [9,10]. Therefore, further investigation is required concerning the contribution of the different stakeholders involved in sustainable food systems (SFSs).

There is still a lack of research in this field. One of the few existing studies analysing stakeholders’ perspective involves EU agency representatives and researchers [6]; nevertheless, it ignores policy makers, businesses, and civil society at the national and regional levels. This study finds that existing policy focuses mainly on food producers and consumers, while neglecting retailers. It also demonstrates low participation of food producers in policy making. The other available studies have a number of limitations. One study [11] examines various local, national, and international stakeholders but only analyses written public communications (e.g., food advertisements or articles from specialised periodicals). Domingo et al. [12] emphasize the connection between food security and SFSs from the perspectives of local community leaders. However, their study only touches upon the issues of how the sustainable food system is understood or what its challenges are. Another study [13] analyses the challenges and successes in developing a local sustainable food system, but it is limited to one country (Australia).

The current study aims to address the aforementioned research gaps by discussing the drivers of, barriers to, and policy recommendations for developing sustainable food systems (SFSs) in four European countries representing different economic, geographical, and cultural contexts.

Our study objectives are threefold:

(1) identify the dimensions of food sustainability addressed in the four involved countries;
(2) analyse the main drivers of and barriers to developing SFSs;
(3) analyse and propose common and specific national solutions for developing SFSs.

To reach these objectives, we analyse national policy documents related to food and sustainability and conduct interviews with various groups of stakeholders in four European countries (Germany, Italy, Norway, and Romania).

Our research objectives are in line with the holistic SFS approach proposed by [9,14], which suggests considering the interconnections between food system members and SFS components, as well as the whole system. For this reason, unlike previous studies [6,10,12,13], this research investigates both national policy documents and stakeholder perspectives on SFSs in a multi-country comparison. The selected stakeholders represent public actors and non-profit and commercial organisations, which is a desirable combination for collaborative SFS efforts [8]. Overall, this paper provides new knowledge on the European national SFS attempts and, by comparing them, lays a foundation for the development of a coherent SFS policy framework.

The paper has the following structure. First, we provide a brief overview of the theoretical perspectives on sustainability-related issues in the food system. Then, we present our methodological approach and the main findings on national SFS policy discourses and stakeholder perspectives. Based on this, we discuss policy implications and recommendations. Finally, we indicate the limitations of this study and suggest future research directions.

## 2. Theoretical Background

### 2.1. Food Sustainability and SFSs

Environmental issues on a larger scale were first acknowledged in the United States in the 1950s, when decision makers and the public had to reflect on the negative environmental impact of economic practices due to a series of scandals related to the use of chemicals in agriculture [15]. The concept of sustainability, which highlights the importance of nature for the socioeconomic system as a result of constant population growth and limited resources, emerged in the 1970s [16], while the notion of sustainable development was first mentioned in 1987 [1]. Although sustainability encompasses three acknowledged pillars—economic, social, and environmental— Béné and colleagues [17] argue for a larger focus on the environmental dimension.

The concept of SFSs appeared in the 1980s and addressed the negative impact of agricultural practices on the quality of food and human health [18]. In the 2000s, the SFS took its current form, representing a socially accepted, holistic, and adaptive complex food system that focuses on achieving sustainability [14,19].

According to the Food and Agriculture Organization [20] (p. 1), an SFS ‘delivers food security and nutrition for all in such a way that the economic, social and environmental bases to generate food security and nutrition for future generations are not compromised’. Moreover, an SFS ‘is one that contributes to all three pillars of sustainability in a balanced manner, and requires the system to be fair’ [6] (p. 31). Additionally, an SFS should focus on food security and safety, sustainable and healthy diets, trade-offs, multi-actor acknowledgement, feedback loops, complexity, and resilience to shocks [17].

To summarise, an SFS consists of an efficient, balanced, and fair system of production, distribution, consumption, and disposal of food based on the three pillars of sustainability (environmental, social, and economic) and the interactions and collaborations between different stakeholders.

Many studies address the consumption side of SFSs by examining individual factors that influence sustainable food choices (e.g., consumer preferences, personal beliefs, and willingness to pay) [21,22,23,24,25]. The majority of the studies indicate that knowledge [26] and price [27] are the most common factors influencing consumer preferences for sustainable products. However, there is a need to consider a wider range of determinants of sustainable food choices—individual factors being only one of them. Macro and structural causes of sustainable consumption are considered even more important than individual-level attitudinal variables for the transition towards SFSs [28]. According to Kearney’s study [29], urbanisation, trade liberalisation, and transnational food retailing have contributed to unsustainable food consumption. Moreover, Bricas et al. [30] address the role of cities in supporting rural communities for developing SFSs, through investments, collaboration policies, local market development, and public procurement from rural communities’ nearby cities. Furthermore, the societal context and policies influence the transition towards SFSs through education, infrastructure, and regulations [23].

Production is another important SFS element. Most studies focus on different topics related to agriculture [31,32], and only a few have examined the role of industrial producers [33]. Several studies address specific drivers, such as food waste valorisation in manufacturing, biosensors, nanotechnologies, innovation, and information technology [34,35,36,37,38]. Additionally, other studies [12,13] point out the importance of locally produced food for ensuring food security and sustainability of the food system.

Few studies approach sustainability from both the production and consumption perspectives to identify the best solutions for SFS development. For example, Lorenz and Veenhoff [39] highlight the role of solidarity and consumer empowerment in stimulating changes in production methods, while Allen et al.’s study [40] points out the need to rebalance the price of unsustainable food with its true costs, which include the negative effects of agrifood practices on the environment.

In addition, few studies focus on the distribution system as part of an SFS. Existing studies present solutions related to energy consumption, carbon footprint, and cost or time efficiencies [41,42,43,44].

Furthermore, food waste management is considered one of the solutions for developing SFSs. For instance, some studies focus on methods of food disposal [45,46], while others analyse the costs attributed to food disposal [47,48] and alternative solutions such as food sharing and donation [49,50].

Overall, many studies on SFSs have recently emerged. However, only a few address the concept of SFS and suggest guidelines for its development [6,26,40]. There is also a need for more research focusing on various stakeholders involved in the production, distribution, consumption, and disposal of food, as suggested by the SAPEA report [14].

### 2.2. Policies for SFS Development

In the EU, the European Commission [4] emphasises the need to implement changes in several food-related areas, such as education, research, innovation, finance, and corporate social responsibility. This calls for more integrated food policies.

Similarly, De Schutter et al. [5] identify several areas for policy improvement, such as coherence across policy areas and governance levels and increasing food democracy. According to these authors, EU policies are ill-equipped to support local ‘alternative food system’ initiatives such as community-supported agriculture schemes and local sourcing for school canteens. There is a need for multi-level governance promoting collaboration and practice-sharing, as well as further support for inclusive, bottom-up initiatives. Food policies should also have an integrated long-term perspective addressing coordinated shifts across the whole food system. Although some tools for SFS development exist (e.g., food schemes and food education), these have not had the desired effect [51]. More information and knowledge about food should be available to stimulate better consumer choices and increase awareness around their consequences: for instance, communicating environmental footprints to consumers through labels or raising consumers’ awareness about food-related emissions [52,53]. Moreover, giving a default choice of sustainable food in different events or places, through nudging techniques, seems to be considerably effective for pushing the consumer to choose sustainable food. For example, an experimental study [54] conducted during three conferences showed that the participants were more inclined to opt for the vegetarian buffet instead of the non-vegetarian choice, if the vegetarian menu was the standard lunch, i.e., the default choice. Additionally, another study [55] conducted on a university campus indicates the positive effect of the nudge in choosing the non-meat food option, by paying attention to the existence of a sustainable default lunch, the default menu configuration, and gender preferences.

There is also a growing focus on how to integrate nutrition and health-related aspects into the common agricultural policy (CAP) [56]. Examples of possible policy solutions in this regard include fiscal measures and restrictions for unhealthy foods, nutrition education in schools, and nutritional labelling [51]. In terms of environmental issues, Recanati et al. [51] emphasise the need to align policy objectives with actions and to consider context and resource particularities through diversified measures. Moreover, Baldy [52] raises concerns about the economic security of small agrifood businesses due to strict EU certification processes, which make large companies more competitive. A reduction in bureaucracy can help address these issues. The literature also acknowledges the role of researchers [51]. For example, the development of innovative sustainability metrics can enable SFS actors to better assess their sustainability-related actions [10].

In addition, several studies highlight the importance of increasing collaboration between different stakeholders at all levels of the food system [7,9,12]. Moschitz’s study [7] proposes stronger involvement of the civil society to achieve a coherent sustainable food policy.

Furthermore, the collaboration between urban and rural areas for SFS development is emphasized in the literature [30] by pointing out the need for local and regional policy development to sustain the rural areas around cities. These could lead to accessible locally produced food for the city inhabitants, lower transportation costs, and higher incomes for farmers, reducing inequalities within the urban population and ensuring food security [30].

## 3. Materials and Methods

This study focuses on four European countries (Germany, Italy, Norway, and Romania) that represent different conditions in terms of geography, socioeconomic situation, environmental performance, and sustainability orientation. Norway, representing Northern Europe, had the highest gross domestic product (GDP) per capita in the sample (EUR 69,770 in 2019). Germany, representing Western Europe, had a GDP per capita of EUR 35,970 in 2019, which was over half of Norway’s GDP. Italy, representing Southern Europe, had a GDP per capita of EUR 26,860 in 2019, which was EUR 9000 less than Germany. Romania, representing the former Central and Eastern Communist Bloc, had a GDP of EUR 9130 per capita in 2019 [57]. Concerning their focus on sustainability, the four countries are also very different. Norway is the only country in the sample that has a reserve of biocapacity, while the other three countries have a debt in biocapacity (calculated as the difference between biocapacity/person and ecological footprint) [58]. In addition, Norway and Germany are ranked 9th and 10th in terms of the environmental performance index, which assesses national environmental health and ecosystem vitality. Italy and Romania are ranked 20th and 32nd, respectively, of 180 countries [59].

Our methodological approach involved two parallel stages of analysis. Firstly, we analysed the national policy documents on SFSs in each country by using critical frame analysis. Secondly, we conducted in-depth interviews with various national stakeholders representing policy makers, agrifood businesses, and civil society, and we analysed them with the help of NVivo and MaxQDA software. Both critical frame analysis and content analysis allowed us to extract key themes, barriers, and solutions proposed for the development of SFSs.

### 3.1. Analysis of National Policy Documents on SFSs

The objective of this analysis was to identify SFS-related issues addressed by the main national policy documents, as well as proposed solutions. The document analysis included two steps: document selection and critical frame analysis.

In the first step, we selected the most representative policy documents related to SFSs in each country. We searched through legislative texts, national strategies, political plans, parliamentary debates, political speeches and declarations, and party programs. The search words included the following: sustainability, sustainable, food, green, environment, organic, and health. The search was carried out in April 2019. Documents were added to the list until they provided no additional information or there were no more documents to add. After skimming through the documents, we selected those that focused on aspects related to both food and sustainability, even if, in some cases, the latter was mentioned only in the background. We ended up with a list of 15–20 documents per country. To ensure diversity within each country, as well as balance and comparability between the countries, we selected the 10 most important documents in each country based on the following criteria: document’s relevance to national policy in the context of food and sustainability; diversity in terms of the type of document; and diversity in terms of topic (Table 1).

In the second step, the 40 selected national documents were analysed using critical frame analysis, a widely acknowledged approach for analysing policies on health, ethics, and food-related topics [60]. This approach provided the structure to policy text exploration, and therefore, it contributed to identifying and comparing problems and solutions in the four different analysed countries.

The critical frame analysis was conducted by following two phases.

In the first phase, we thoroughly read the texts of all 40 documents and synthetised the information in the four national languages, based on a supertext template developed by [61,62]. Each template presented information about the text in general, as well as the voice, diagnosis, prognosis, normativity, balance, and further comments (see Table A1 in the Appendix A).

In the second phase, the 40 supertexts were translated into English, and we then identified the main issue frames in each document and compared the documents investigating the same issues within each country and across countries in line with the research objectives. All five authors of this paper were directly involved in conducting this analysis, while other members of the research project supervised the process. Researchers exposed the frames in the policy texts to explore discourses, context, topics, representations, coherency, inconsistencies, and normativity and to identify problems and solutions in policy debates [63,64].

### 3.2. In-Depth Interviews with National Stakeholders

The objective of the in-depth interviews with national stakeholders was to explore their opinion on SFS development in each country.

To achieve a heterogeneous set of informants, we selected different stakeholder groups: policy makers at the national and regional levels, agrifood producer associations, institutions responsible for food certification, agrifood consultants, consumer associations, environmental associations, health associations, cultural associations, non-governmental organisations (NGOs), and researchers (Table 2).

The informants interviewed per country had a professional background, experience, and knowledge related to SFSs. The national sets of informants were selected to ensure comparability between the countries. We interviewed ten representatives of the different stakeholder groups in each country.

The interview guide was first developed in English and then translated into the respective languages using back-and-forth translation. The following topics were addressed: the concept of food sustainability and SFSs; drivers of and barriers to SFS development; the role of regulations, policy, education, infrastructure, and technology; the role of SFS actors; and future perspectives. We used a semi-structured interview approach by asking non-exhaustive, open, storytelling, and probing questions to encourage the dialogue.

The interviews were carried out in April to September 2019. They were recorded and transcribed in national languages. All five authors of this paper were directly involved in conducting the interviews and analysing the transcripts; other members of the research project collaborated in conducting interviews, transcriptions, and content analysis. We used the constant comparative method [65] for text analysis, which is based on the following steps.

In the first step, the content analysis was facilitated using NVivo and MaxQDA software [66]. In each country, the interview transcripts in the national language were transferred to one of these two software applications.

In the second step, at least two coders read each interview text in each country and coded the text by using the software. The first coder created initial coding categories that reflected the consistencies and main themes emerging in each text. The second coder audited the text, paying careful attention to those areas that the first coder identified as exemplary responses that illuminated the emergent themes [67]. The coding categories were created in English to allow comparability across all four analysed countries.

In the third step, the categories and themes were compared within the same interview and between interviews [68].

Lastly, the coded categories were analysed and compared in English between the four countries to identify main common and specific themes related to drivers and barriers, as well as solutions for developing SFSs.

## 4. Results

**Figure 1:**
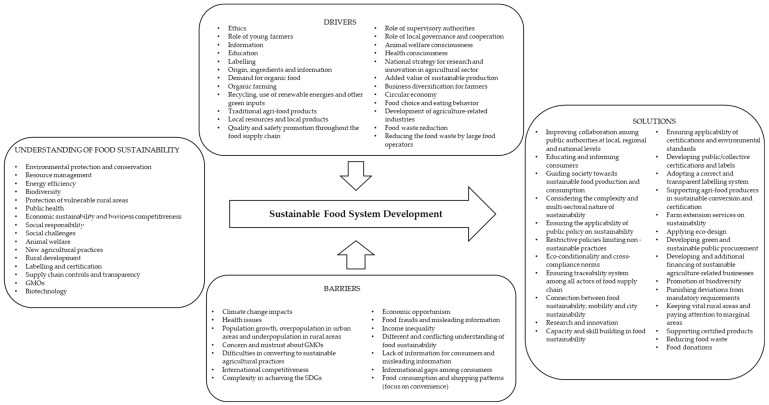
Understanding of food sustainability; barriers to, drivers for, and solutions for SFS development identified in policy discourse in the four investigated countries.

**Figure 2:**
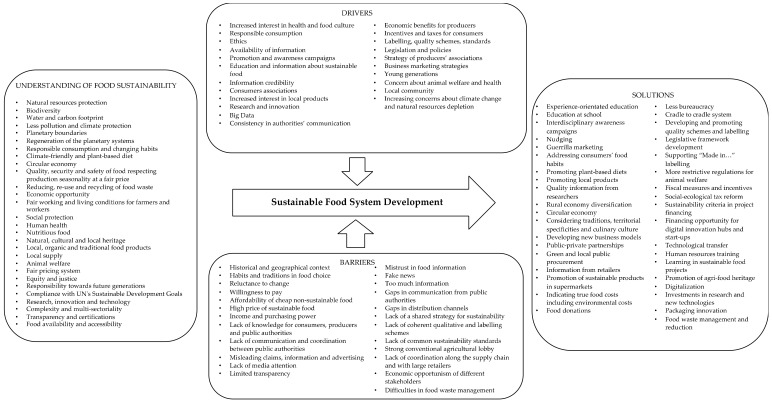
Understanding of, barriers to, drivers for, and solutions for SFS development identified by stakeholders in the four investigated countries.

The findings of the policy discourse and stakeholders’ perspective analysis are synthetically illustrated in Figure 1 and Figure 2.

### 4.1. Dimensions of Food Sustainability and SFS

Some dimensions of food sustainability receive considerable attention from both national policies and interviewed stakeholders in all countries (see Table A2 and Table A3 in Appendix A).

Most of the analysed policy documents focus on the social dimension of sustainability (e.g., D8, D16, D25, D31): on issues such as public health, food safety, correct product information for consumers, and consumer education. However, stakeholders differently approach the social aspects of sustainability in the four countries. Norwegian stakeholders (S23, S29) argue that food sustainability goes beyond reducing emissions and pollution and includes such social aspects as the working and living conditions for employees in the food industry. Some German (S7, S9, S10) representatives further emphasize the social dimension in the whole value chain. German and Norwegian stakeholders (S10, S21–S24) also address the nutritional aspect of sustainable food (e.g., hunger, obesity, affordability, and healthy diet). Food availability, in terms of access and affordability, is a requisite for food sustainability for the Romanian experts (S31, S36, S39). Some Italian informants (S11, S17, S20) highlight the importance of recovering traditional food heritage to generate positive spillovers at both production (e.g., biodiversity conservation and local know-how) and consumption (waste reduction and efficient use of natural resources) levels. One Romanian stakeholder (S32) also emphasises the need to preserve the local food heritage as part of developing an SFS.

Another common dimension of high interest in the four countries is the environmental dimension, e.g., protection of environment, biodiversity, and vulnerable ecological areas. Meanwhile, most stakeholders associate the SFS with the environmental dimension of sustainability. Some stakeholders focus more on eco-labelling (especially the German ones—e.g., S1–S3), protection of natural resources and biodiversity (Italians—S18 and S20), pollution and food waste (Norwegian stakeholders—S22–S30), and, in general, environmental protection and conservation (almost all Romanian interviewees) and planetary boundaries (Germans and Romanians—S4, S6, S36).

However, we also find variations between the countries in terms of addressed topics. In the German policy discourse, there is a strong focus on environmental protection (e.g., soil and water protection and air pollution) and the labelling of organic products, as well as food and resource waste (e.g., D3, D4, D6). In Italy, there is a greater emphasis on the economic dimension of sustainability, e.g., the transition of the whole economic system to a green economy and promoting various territorial collaborations (e.g., rural district, green community—e.g., D12).

In Norway, the focus is on animal welfare, with seven out of ten documents mentioning this issue (D22, D25–D30), and on sustainable growth for aquaculture both in economic and environmental terms (e.g., D21). In Romania, the focus is on food production, labelling rules, and organic agriculture.

Only a couple of Italian and Norwegian informants (S12, S15, S24, S26) discuss the economic dimension of SFSs, while both German and Romanian stakeholders (S7–S8, S33–S34, S37) emphasise that food producers and consumers will only act if it is economically viable for them.

### 4.2. Drivers for and Barriers to Developing SFSs

One of the main drivers and barriers towards SFS development emerging in both policy discourses and stakeholders’ perspectives for all countries is the need for further understanding and knowledge of how various actors (i.e., producers, intermediaries, retailers, consumers) can contribute to sustainability (e.g., D1, D12, D27, D32, S2, S9, S12, S26, S34). Additionally, according to the stakeholders, the distribution of the right information is another common driver. As pointed out by the Norwegian stakeholders (S26), a lot of information about sustainable food comes from private individuals (e.g., bloggers, YouTubers) instead of governmental officials. Furthermore, most Italian stakeholders highlight the poor quality of information and the lack of reliable institutional information, while German experts mention both the lack of consumer awareness and misleading claims (e.g., S2, S3, S9). Similarly, in Norway, the stakeholders discuss the confusion around the concept of sustainable food (e.g., S22–S24, S26). They (e.g., S26) argue that there is a need to adjust the definition of food sustainability according to local conditions, e.g., it might be sustainable to produce dairy products if the agricultural land cannot be used in any other way due to natural constraints. Additionally, one Romanian expert (S36) emphasizes the negative effect of the conflicting messages shared through the Internet and the lack of positive role models. The communication is often emotionally driven, and the risk is that people end up with a skewed concept of food sustainability (S26). Additionally, there is an incorrect use of information on food products’ labels (D33, D39), i.e., missing, incorrect, and abusive use of information, especially for organic products.

According to both the documents (e.g., D1, D13, D24, D31) and the interviewed stakeholders (e.g., S9, S16, S24, S33), the role of consumers is recognized in the SFS in all four countries; the need for guiding consumers towards a sustainable food choice is mentioned. Additionally, considering the stakeholders’ opinions, what consumers acknowledge as good food may also be an issue. For instance, Norwegian stakeholders (S22, S26, S28) highlighted that consumers need to learn to eat the whole animal and be less critical of expired food to reduce food waste. In contrast, in Romania, the focus is still on developing the infrastructure for food waste management (S31), while consumers’ reluctance to change (S31, S36) and ‘living in the moment’ attitude (S32) are seen as other relevant barriers. Furthermore, some stakeholders (e.g., S1, S17, S22, S36) believe that established food habits and a lack of time prevent consumers from making more sustainable food choices. The age of consumers is another driver. Younger generations can more easily adapt to new sustainable food habits, while older generations have problems changing their food consumption. As discussed by German informants, ‘habits to buy and cook what has always been bought and cooked’ makes the transformation more complicated. Food is a sensitive and individual product, and people do not like to be told what to eat. Norwegian stakeholders also agree that, to a large degree, habits and traditions shape consumer diets and create resistance to change. Meanwhile, culture and history strongly influence consumers’ mentality (e.g., Romanian consumers are used to buying large quantities of food as a result of food deprivations during the communist era). This barrier should be taken into consideration when developing communication strategies for consumers.

Although it is not mentioned in the policy discourses, another common driver and barrier for stakeholders is the availability of sustainable food, in terms of access and affordability. Firstly, in Germany, although the selection of sustainable food—mainly organic-certified—has been steadily increasing, several stakeholders highlight the problem of easy access to sustainable food in retail shops, restaurants, and cantinas (S1, S3–S5). Romanian sustainable food initiatives are limited to only a few types of organic food, which are only sold in supermarkets or online (S34, S39–S40). However, many Romanians obtain their food directly from farmers and their own family gardens, which makes the local supply chain important for gaining access to sustainable food. Norwegian stakeholders (S22, S27, S29) complain about the poor selection of sustainable food in their supermarket chains and blame their limited access on the power of retailers. Similarly, Italian stakeholders (S14–S15) highlight the retailers’ role in influencing consumers’ choices through their store assortments and sales strategies, and they (S16–S19) call for fairer procurement strategies and greater efforts in logistics and product packaging (e.g., reducing the use of plastic packages). Secondly, several stakeholders (e.g., S3–S4, S9–S15, S27, S29, S30, S32, S36, S39) consider the affordability of sustainable food as an important barrier and mention the negative effect of high prices on the demand for sustainable food, which is seen as a luxury by Italians and Romanians. German, Italian, and Romanian stakeholders also highlight the higher cost of producing and marketing sustainable food, which, however, will decrease as sustainable production practices become more mainstream.

Finally, technology, research, and innovation are acknowledged as common drivers of SFS development in both policy discourses and stakeholders’ perspectives (e.g., D20, D35, S5, S13, S18, S20, S27, S36, S37), as they ensure food safety, productivity, economic efficiency, lower environmental impact, better control and information, and higher user convenience.

Some drivers and barriers receive special attention in each country.

In the Italian policy discourses (D12, D17) and German and Romanian interviews (S6, S33), another concern is the competitiveness of national products on international and national markets. Furthermore, German and Romanian informants (e.g., S7–S8, S40) discuss unfairness in terms of sustainable food labelling and production (e.g., extra labelling and production costs that organic producers have to bear while conventional producers do not).

The German documents (D1, D4–D6) identify overusing the Earth’s limited resources as a general problem and emphasise the great potential of household consumption to reduce environmental impacts and food waste, while German stakeholders (e.g., S1, S3) mention the strong conventional agricultural lobby as a challenge towards SFS development.

The Italian documents highlight (D11–D13, D15, D17, D19) the need for a different economic model that focuses on saving natural resources; providing quality food products in terms of safety, health, and environmental protection; and supporting vulnerable producers and rural areas. While Italian stakeholders (S14, S18–S19) identify the lack of a common set of criteria defining sustainable food as a major barrier that leads to several challenges, including proliferation of private certification schemes that are mainly implemented by large producers, the role of certification for consumers’ food choices is marginal, and the interest among retailers to increase the availability of sustainable food is low. Moreover, some Italian stakeholders (S12–S13) claim the oligopolistic power of food retailers, who, paradoxically, continue to adopt unsustainable practices in food procurement, logistics, and packaging, despite producers’ investments in sustainability (e.g., in the case of fruit and vegetable producers).

The Norwegian policy documents (D22, D23, D25-D30) identify a large number of sustainability-related issues as important drivers for building an SFS, including animal welfare; the labelling of organic products and its misuse; the dangers of using genetically modified organisms (GMOs); and the overpopulation of urban areas, leading to reduced development of rural areas and agriculture-related industries. A Norwegian stakeholder (S27) believes that Norwegian food safety regulations prevent the use of leftover foods and therefore lead to more food waste. He also mentions the lack of marketing skills among Norwegian food producers, which complicates the promotion of local sustainable food. The Romanian documents consider the context of sustainable development as having a more voluntary rather than compulsory character. They also identify the problems related to eco-conditionality compliance (i.e., granting funds in exchange for good environmental practices in D31 and D38), food labelling, food waste, and controlling and reducing the use of pesticides in agriculture for pollution and health-related reasons (D32, D36). Romanian stakeholders (e.g., S37–S39) address the absence of a consolidated legislative framework and national strategy for SFS development, food security, and food waste along the supply chain; the lack of interest towards sustainability among food distributors; and the misalignment of economic interests between various actors in the food system (S31, S36). This results in limited administrative facilities and a lack of support and incentives to stimulate interest for sustainable food among both producers and consumers.

### 4.3. Proposed Solutions for SFS Development

The national policy documents and the stakeholders suggest a number of common solutions for SFS development for all four countries.

A common general solution is to increase collaborations in the food system (e.g., D9, D14–D15, D23, D32, D35, S2, S11, S24, S39). Additionally, the stakeholders discuss various solutions for improving collaboration between different actors. Thus, it is crucial to encourage a variety of different actors to join in, with authorities taking on a coordinator role (e.g., S8, S18, S24, S31). Italian stakeholders (S11, S14) suggest organising different ‘discussion tables’, while Romanian informants (S31, S32, S39) propose creating a national rural development network and associations for sustainable producers. The extended collaboration between different SFS actors will allow the authorities to use one voice to communicate food sustainability, which is an important success condition according to the interviewees (S8, S15, S19, S22, S39, S40).

Another common general measure for developing the SFS is providing correct information for consumers (D1, D19, D24, D38, S1, S11, S24, S38).

In addition, the stakeholders discuss concrete actions required for improving the education and information for consumers and other actors. For example, many stakeholders agree that extended communication efforts are required to increase consumers’ interest in sustainable food—e.g., by including sustainability in the school curriculum (e.g., S8, S15, S28, S32). While communicating with consumers, authorities need to take the lead (S2, S4, S18–S19, S24, S33) and use one voice (S11, S22) to avoid confusion. At the same time, the majority of the German stakeholders (S2, S5) very strongly recommend that public authorities also provide clear and uncomplicated information about sustainable food to producers. They also need to focus more on the environmental dimension of sustainability to bring young consumers on board (S3–S4, S15, S17), refer to scientific studies (S11, S15), and enlarge the concept of sustainable food beyond organic food (S11–S12, S15–S17). Some German stakeholders (S2, S7) also suggest softly influencing (‘nudging’) consumer choices instead of imposing new rules like banning junk food. In addition, learning projects (e.g., comparing an organic field with a conventional field) can help both adults and children to understand the impact and consequences of their decisions and actions on the environment (S6). Italian, Romanian, and Norwegian stakeholders (e.g., S15, S28, S36) also mention the importance of influencers, role models, and positive examples in media communication, especially for promoting sustainable food to younger generations. Some Italian and Romanian stakeholders (e.g., S17, S36) believe in ‘innovative’ communication tools such as apps, ‘smart labels’, transparent labels, and bilateral communication between businesses and consumers.

Likewise, stakeholders argue that distributors have substantial power to influence consumer choices—even simple measures such as product placement can make a big difference. Some stakeholders (e.g., S3, S12, S25, S32) emphasise the role of public procurement in encouraging sustainable food practices. Additionally, Norwegian experts (e.g., S22–S23, S27) want grocery chain stores to take more responsibility by offering and promoting more sustainable foods and explicitly explaining the consequences of choosing or not choosing those foods. One Romanian expert (S34) emphasises the need to develop dedicated sustainable shops and recycling infrastructure in shopping areas. Some Italian stakeholders (S18–S20) address non-sustainable transport and logistics systems. At the same time, German and Italian stakeholders (S8, S16) further argue that consumers can have a strong influence on retailers by expressing their preferences, while retailers may influence producers. For example, an Italian stakeholder (S14) highlights the importance of involving retailers in sustainability discussions and certification.

Additionally, food producers need to be honest and transparent regarding how food is being made, and they should offer certified high-quality products (S3, S7, S26, S29, S32). However, high food quality can also be an issue because it makes food less affordable. To address this issue, Romanian and Norwegian stakeholders (e.g., S27, S28, S33, S40) suggest providing benefits to sustainable food producers—e.g., by reducing taxes and serving their food at all public events—and considering higher taxes on unsustainable food. Several German interviewees (e.g., S1, S9) point out the importance of taxation reform for sustainable food production—e.g., by introducing CO_2_ taxation. To make consumers aware of the true costs of food products, German and Italian stakeholders (S8, S15) suggest showing true cost prices at the point of sale.

Both the analysed policy documents and stakeholders acknowledge another general need: that of further developing the regulatory framework through establishing standards of sustainable food production and labelling schemes (e.g., D2–D3, D15, D26–D30, D33, S10, S15, S18, S21, S29, S34, S36).

Specifically, on the one hand, the prevailing solution in the Norwegian policy documents (D22, D25–D30) is strengthening the authorities’ control in food production and its related activities, such as the holding of animals. The documents mainly focus on setting the rules and requirements for the various actors in the food system to ensure food safety, sustainable development, and adherence to ethical concerns. Control is also an important part of the Romanian policy solutions (D32, D34, D37), especially concerning human health and food safety. On the other hand, from the perspectives of stakeholders, the Germans (S5, S10) wish for a change to the standards for animal welfare, while the Norwegians (S27, S29) call for more laws and regulations regarding animal feed, animal products, and food waste. Additionally, it is important to have a national strategy on SFS development with clear objectives, together with a national plan with mandatory implementation and clear indicators, as suggested by Romanian and Italian experts (S11, S14, S32, S36, S39). Moreover, as recommended by one of the Norwegian stakeholders (S24), a sustainable food policy should cover various target areas, such as district/rural, agricultural, and industrial policies.

Both the analysed policies and stakeholders focus on finding solutions to diminish or limit humans’ negative impacts on the environment, such as implementing better food waste management (D4, D13, D36, S6, S13, S23, S24, S25, S34). Public authorities and consumer associations (S2, S8, S18–S20, S25) believe that greater efforts should be made at the distribution level, in terms of increasing food shelf life, recycling, reducing food waste, and organising food donations, while policy documents in all four countries (D1, D14, D22, D35) point out the importance of increasing awareness about biodiversity.

In addition, considering the social dimension in particular, the German documents (D1, D6) suggest that people should lead a sustainable lifestyle. Moreover, the perspectives of German and Norwegian stakeholders (e.g., S1, S3, S7, S25–S27) on health, nutrition, and diets emphasise consumers’ need to adjust to a healthier and more sustainable diet that consists of more fruit, vegetables, and whole grains and less meat, especially in school cantinas and public institutions.

From the perspective of Italian policies (D11–D12, D17), it is important for society to include environmental and social considerations in public decision making to favour the transition to a green and circular economy. However, the documents mostly provide the general principles of this transition, with limited practical applications (D17). An implemented solution is the regulation on green public procurement in order to stimulate sustainable production practices along the supply chain through the national and regional public administrations (D16). Additionally, according to German documents (D3, D9), there is a strong effort to increase the share of organic food in the agricultural sector by introducing a coherent legal framework, improving access to organic farming, making use of and expanding existing demand potential, improving the performance of ecological agricultural systems, and rewarding environmental services. Furthermore, Norwegian stakeholders (S21, S25, S27–S29) believe that consumers should be encouraged to buy more locally produced food by reducing taxes on local food, supporting local farmers, and increasing marketing efforts for local food.

Another important general solution from the point of view of stakeholders is the development of technologies, infrastructure, and innovations for creating SFSs. Thus, stakeholders (S2, S8, S11, S17–S20, S24, S26–S27, S30, S36) argue for the increased use of sustainable innovations and technology throughout SFSs (e.g., aimed at carbon- and water-footprint measurements, packaging, product shelf life, food waste, improving digitality). All Italian stakeholders agree that technology can help food producers improve their sustainable practices (e.g., big data in agriculture or nanotechnology for packaging and product shelf life). The regional Italian authorities (S18–S19) particularly highlight the relevance of entrepreneurial skills in terms of innovation propensity and cooperative business models.

There is also a need for infrastructures such as developing ‘incubators’ and digital platforms where different SFS actors can exchange ideas and knowledge, improving food waste disposal, especially in Romania (S34, S36, S39). Furthermore, German and Italian experts (S5, S16, S18) suggest increasing public investments in research for developing practical sustainable solutions, while Norwegian informants (e.g., S28) stress the need for increased knowledge on biological processes, ecosystems, and agriculture in general, because it is important to have a more research-based approach and to test sustainability-related measures before implementing them on a large scale. Similarly, Romanian stakeholders (S32, S36, S39) suggest establishing professional associations on a global level to analyse risks in the food system.

Furthermore, we find country-specific policy measures. For example, the Italian policy documents relevant for the agrifood sector (D13–D15, D18–D20) include product traceability, financial support for environmental initiatives, biodiversity protection, collaboration networks, and research and innovation. In addition, some Norwegian policy documents suggest measures for achieving sustainable growth in both economic and environmental terms (D21) and further development of rural areas (D23). Other Romanian solutions from the policy discourses aim to reduce the use of pesticides (D32, D37) and reassess the production process to become organic (D31).

## 5. Discussion

Our results indicate that all pillars of food sustainability (environmental, social, and economic) are perceived as crucial for further SFS development in the four selected countries, despite some variations in importance. Moreover, there is a need to reach a consensus on the definition and understanding of food sustainability, as was also observed by [14,17]. However, similar to [69], we acknowledge the importance of the national context while providing the recommendations.

Based on the analysis of the common drivers and solutions in the national policy documents, we identify the following existing measures for SFS development. First, the analysed documents in all four countries highlight the need to better understand different actors’ contributions to food sustainability and increase collaborations in the food system, as this can improve the regulatory framework and competitiveness of local products in national markets [29]. For example, a measure proposed in the Italian documents is to develop collaboration networks, as also discussed by [14]. Second, national policies aim to increase consumer awareness about biodiversity and provide correct information about sustainable food, which can help consumers to make better-informed decisions [52]. Third, measures of environmental protection (e.g., reducing overexploitation and food waste, using renewable energy) ensure food sustainability, as also indicated in previous studies [9].

Additionally, the country-specific measures suggest that rewards or financial support are provided for environmental practices in Germany, Italy, and Romania. Moreover, in Italy and Norway, the policy documents focus on vulnerable producers and the development of agriculture-related industries in rural areas. Italian policies emphasise the need to ensure product traceability and green public procurement [41]. The Norwegian policies focus on ethical and sustainable principles in food production, especially in relation to animal welfare, similar to [70]. Better control for food safety and quality (e.g., GMO, pesticide use) is addressed in Italy, Norway, and Romania.

Despite the existing measures discussed above, the transition towards SFSs requires additional policy efforts. Based on our interviews with different types of national stakeholders, we identify several gaps in the existing national SFS policies and indicate how these gaps can be addressed.

First, the stakeholders in all four countries propose several additional measures regarding consumers’ education and communication, similar to the previous studies [13], but with some differences in the particular sub-themes of the results. For example, on the one hand, while our results consider the involvement of all actors in communicating about sustainable food, Sambell et al. [13] focus specifically on farmers, researchers, and local communities. On the other hand, a similar solution is to improve the producers’ knowledge about food products. Furthermore, the informants in all four countries recommend that both private individuals and governmental officials provide clear and consistent messages on sustainable food, as also suggested by Blay-Palmer, Sonnino, and Custot [26]. In addition, they advise developing labelling standards for sustainable food, similar to the study by Vanham and Leip [53], as these are inadequate [13]. Furthermore, they propose introducing the sustainability debate into the school curriculum, similar to Allen et al.’s study [40]. German stakeholders suggest providing clear information about sustainable food to producers [13], while Norwegian stakeholders argue that local food producers should develop better marketing skills to make sustainable food more attractive. Moreover, as previously discussed [71], Italian, Norwegian, and Romanian stakeholders believe that sustainable food marketing should focus on this food’s environmental and health benefits, high quality, and altruistic attributes (e.g., animal welfare). They also recommend involving influencers and positive role models and using ‘innovative’ communication tools such as apps and ‘smart labels’. Additionally, German stakeholders suggest using nudging techniques.

Second, despite the existing policy measures, both stakeholders and recent studies [6,14] argue that improving stakeholders’ collaboration is still a desirable objective. The stakeholders argue for the importance of further collaborations between different SFS actors. This will allow the authorities to use one voice to communicate food sustainability, which is an important success condition according to both the interviewed stakeholders and previous research [72]. As demonstrated earlier [73], the most effective and trustworthy way of providing information on sustainable food is through the involvement of several actors, e.g., when producers’ unions communicate environmental benefits, health experts communicate health benefits, and public authorities communicate social benefits.

Our findings add several concrete examples of how to involve different actors in developing SFS policies, similar to Moschitz’s study [7]. For example, Romanian stakeholders suggest developing collaborative networks of agrifood stakeholders, whereas Italian stakeholders suggest organising discussion tables. In Norway, they propose inviting different actors to debates focusing on SFS initiatives and measures, similar to Gruchmann et al.’s study [74]. However, public authorities should take the lead role in this process. They should also change and consolidate the current regulations and standards for SFS development [6].

Third, the stakeholders emphasise the role of technology, research, and innovation in stimulating the development of SFSs, as also found in the previous literature [6,13,38]. Sustainable policies should cover all aspects of sustainability (e.g., the proximity factor is usually ignored).

Fourth, another valuable recommendation from stakeholders in all four countries is to increase the availability and affordability of sustainable food, as also addressed in previous studies [40,73]. Procurement, distribution and retailers play an important role when speaking about availability, as acknowledged by Italian stakeholders and earlier studies [13,30,74]. Thus, there is a need to introduce several measures, such as developing sustainable public procurement based on local sustainable food, establishing sustainable shops and start-ups for SFSs, visible placement for sustainable food, and better recycling infrastructure and food disposal. Additionally, the affordability of sustainable food could be improved by reducing taxes on local food and, as suggested by Bartolini et al. [41], closing the gap between the prices of unsustainable food and sustainable food (e.g., through a ‘true cost’ policy). The country-specific recommendations focus on supporting local farmers and increasing marketing efforts for local food in Norway and considering higher taxation of unsustainable food in Germany, Norway, and Romania, similar to Bravo et al.’s study [71]. It can also be relevant to financially incentivise Romanian consumers of sustainable food, as high food quality could potentially increase food prices [71].

Fifth, SFSs should also focus on healthy diets. However, there is an ongoing debate in the literature [17] regarding whether a healthy diet is necessarily sustainable. Despite some obvious synergies (e.g., favourable health effect of reducing animal protein in human diets), a healthy diet mainly concerns nutrient intakes, which can be gained from any kind of food, including those foods with high greenhouse gas emissions [75,76]. German and Norwegian stakeholders suggest adopting a truly sustainable plant-based diet due to its health and environmental impacts [14], while Italian stakeholders emphasise the healthiness of a Mediterranean diet based on local food.

Furthermore, the stakeholders recommend reducing the power of conventional agricultural lobbyists in policy development. Additionally, there is a need to reduce food waste in Norway, similar to [50]. It is also important in Italy and Romania to implement a national strategy on SFS development with clear objectives, together with a national plan for mandatory implementation and clear indicators [6,9,13].

Based on our analysis of the stakeholders’ recommendations in all four countries, we argue that the first step in the further development of SFS policies should focus on interdisciplinary and trans-sectorial policy integration and increasing stakeholder collaboration across all sectors of the economy. Policy makers should take the lead in bringing together representatives from each stakeholder group involved in SFSs. They need to ensure higher consumer involvement by providing better information about sustainable food. Providing a coherent message is imperative to increase knowledge about sustainability and SFSs among all stakeholders, including consumers.

To achieve this, the European countries can develop a common platform at the international level, which can be further adjusted to the national context. The platform can gather information about all sustainable policies and practices, such as new labelling systems and support opportunities for SFS stakeholders. It can also be used to analyse and compare various sustainable inputs and processes, which would provide better transparency for consumers and international cooperation. Thus, the platform can facilitate further partnerships between countries and national and international stakeholders at various levels to ensure a more efficient development of SFSs.

Furthermore, we suggest stimulating technological development, research, and innovation for sustainable practices [34,35,36,37,38], e.g., by providing governmental support to research dedicated to new green technologies and the food companies adopting these technologies. We also recommend increased use of sustainable public procurement, which can help to change the default food option to a more sustainable one. These actions could also help to solve the problems related to the availability and affordability of sustainable food, as the new technologies can reduce the costs of sustainable food production and therefore make it more attractive to various SFS actors.

We also identify several country-specific policy recommendations to address the most pressing issues in each country. It is important to address the affordability of sustainable food in Romania and to develop common standards to define sustainable food in Italy, while in Norway and Germany, the focus should be on educating and informing different SFS stakeholders about sustainable food.

Finally, some of the country-specific best practices can be used to develop shared policies and tools. It is important for different countries to learn from each other, as some policies can be transferrable across countries. For instance, while Romania is at the initial stage of sustainable management of fertilisers and pesticides, Italian legislations are already offering future policy trends in this area by promoting biodistricts. This finding reveals the opportunity to skip some stages in the sustainable management of fertilisers and pesticides in Romania by implementing an adapted Italian practice, thereby achieving policy collaboration as indicated by [6,9]. The same opportunity has also been found in the case of public procurement. Italy is regulating green public procurement, and this could be adapted and implemented by other countries according to their particularities (e.g., Norway and Romania). In contrast, Norway has well-developed regulations on animal welfare and food safety that can be adopted by other countries (e.g., Romania). Furthermore, Germany’s well-developed labelling system of organic food and its national network for donating close-to-expired food are valuable practices that could be adopted in other countries.

## 6. Conclusions

Based on the analysis of the national policy documents and the interviews with the stakeholders in four European countries, the current study identifies several important gaps in the existing national policies for SFS development and suggests solutions that can help to overcome these issues. For example, to achieve policy integration and stakeholder collaboration across all sectors of the economy, we suggest introducing an international common platform, which could be adjusted to the national context.

To our knowledge, this is the first study to compare national policies and stakeholders’ opinions on SFS development in a multi-country analysis. The previous literature [14,19] indicates the need to build a holistic SFS and calls for further investigation regarding the contributions of the different stakeholders involved in SFSs. Therefore, we contribute to the theoretical development of SFSs by analysing cross-country stakeholders’ perspectives and comparing them with existing food policies, as well as by addressing local stakeholders and more groups of actors compared with the previous studies [6,10,12,13,14].

However, the current study has several limitations. First, we analyse the national policies of only four European countries. We invite future studies to conduct a similar analysis in other European countries to extend the generalisability of the results. Second, in each country, we focus on ten major public policy documents. Despite our careful procedure for the document selection, further research could extend the document sample. Moreover, it would be interesting to discuss SFS development with a broader group of stakeholders. Finally, considering the need for policy integration emphasised in this paper, collaborations among public actors and other stakeholders should be further explored.

## Figures and Tables

**Table 1:** Selected policy documents in each country.

No.	Document	Year
	Germany	
D1	‘National Sustainable Consumption Program: Social change through a sustainable lifestyle’, SP, Ministry for Environment, Nature Conservation and Nuclear Safety	2017
D2	‘Law on the introduction and use of a label for organic products’, L 66/2001	2001
D3	Strategy for the future of organic farming—impulses for more sustainability in Germany, SP, Federal Minister of Food and Agriculture	2017
D4	‘Guidelines for the Transfer of Food to Social Institutions—Legal Aspects, Unit 216—Sustainable Nutrition, Food Waste Reduction’, ILR, Federal Minister of Food and Agriculture	2018
D5	‘Sustainable nutrition—What our food has to do with climate protection and global nutrition’, Guidelines, Ministry of the Environment, Energy, Food and Forestry Rhineland-Palatinate	2018
D6	‘Organic farming in Germany’, ILR, Federal Ministry of Food and Agriculture	2018
D7	‘The German organic seal—trademark protection until 2021’, ILR, Federal Institute for Agriculture and Food	2016
D8	‘Genetic engineering and food: The most important facts. Questions and answers on the use of genetic engineering in food’, ILR, Federal Ministry of Food, Agriculture and Consumer Protection	2013
D9	‘German sustainable development strategy—Update 2018’, SP, Federal Government	2018
D10	‘The sustainable shopping basket—Chapter 2: eating and drinking’, Guidelines, Federal Council for Sustainable Development	2018
	Italy	
D11	‘Environmental regulation’, Dlgs 152/2006	2006
D12	‘Environmental provisions to promote measures on green economy and reduction in excessive use of natural resources’, L 221/2015	2015
D13	‘Budget Law 2019’, L 145/2018	2018
D14	‘Provision to protect and promote biodiversity of agricultural and food interest’, L 194/2015	2015
D15	‘Guidance and modernization of the agricultural sector’, Dlgs 228/2001	2001
D16	‘Code of public contracts’, Dlgs 50/2016	2016
D17	‘Contract for the government of change’, political programme of a party coalition, 2018–2019	2018
D18	‘Disciplinary regulation for violation of the provisions of regulation EU no. 1169/2011’, Dlgs 231/2017	2017
D19	‘Ratification and execution of the agreement between the Italian Government and WHO’, L 205/2015	2015
D20	‘Strategy plan for innovation and research in the agricultural, forest and food sector 2014–2020’, SP, Ministry of Agriculture	2015
	Norway	
D21	Regulation on capacity increase for aqua culture permissions—salmon, trout, and rainbow trout, R 2017-12-20-2397, Ministry of Trade, Industry and Fisheries	2017
D22	Regulation on ecological production and labelling of ecological agriculture products, aqua culture products, nutrition, and forage, R 2017-03-18-355, Ministry of Agriculture and Food and Ministry of Trade, Industry and Fisheries	2017, last revision 2020
D23	Circular letter on industry development 2011, CL M-1/2011, Ministry of Agriculture and Food	2011
D24	Regulation on food information for consumers, R 2014-11-28-1497, Ministry of Agriculture and Food	2014, last revision 2017
D25	Law on genetic engineering, L 1993-04-02 nr 38, Ministry of Climate and Environment	1993, last revision 2015
D26	Law on food, L 2003-12-19-124, Ministry of Health and Care Services	2003, last revision 2018
D27	Regulation on changes in the regulation on pesticides, the regulation on fees in the food management and the regulation on payment of fees on particular outputs from the Norwegian Food Safety Authority, R 2018-09-17-1501, Ministry of Agriculture and Food	2018
D28	Law on animal welfare, L 2009-06-19-97, Ministry of Agriculture and Food	2009, last revision 2018
D29	Regulation on changes in the regulation on chicken and turkey husbandry, R 2017-03-06-281, Ministry of Agriculture and Food	2017
D30	Regulation on changes in the regulation on cattle, R 2016-06-23-790, Ministry of Agriculture and Food	2016
	Romania	
D31	‘Organic agrifood products and the establishment of measures in the field of organic food products’, OG 29/2014 for the amendment of art. 6 par. (2) of EOG no. 34/2000	2014
D32	‘Establishing the institutional framework of action for the sustainable use of pesticides on the territory of Romania’, EOG no. 34/2012	2012
D33	‘Approval of OUG no. 34/2000 on organic food products’, L 38/2001	2001
D34	‘The national rules on the authorization of imports of organic agrifood products from third party-countries’, MO 51/2010, Ministry of Agriculture and Rural Development and the National Sanitary Veterinary and Food Safety Authority	2010
D35	‘Food safety’, L 150/2004	2006
D36	‘Diminishing food waste’, L 217/2016	2018
D37	‘Cross-compliance rules under schemes and support measures for farmers in Romania’, MO 352/636/54/2015, Ministry of Agriculture and Rural Development	2005
D38	‘Specific rules on the labeling of organic food products’, MO 417/2002	2002
D39	‘Certification of traditional products’, MO 724/1082/360/2013, Ministry of Agriculture and Rural Development	2013
D40	‘National Strategy for Sustainable Development of Romania 2030’, Government	2018

Note: L = Law; Dlgs = Legislative Decree; OG = Ordinance of Government; EOG = Emergency Ordinance of Government; MO = Ministry Order; ILR = Information on Laws and Regulations; SP = Strategy Plan; R = Regulation; CL = Circular Letter.

**Table 2:** Informant type interviewed by country.

No.	Type of Stakeholder Interviewed	Role of Interviewee
	Germany	
S1	National authority	Head of department
S2	Regional authority	Head of department
S3	Certification association	Secretary
S4	Certification association	President
S5	Certification association	President
S6	Consumer association	Managing director
S7	Consumer association	Deputy director
S8	Food producer association	Head of department
S9	Research organisation	Researcher
S10	Research organisation	Researcher
	Italy	
S11	Agrifood consulting firm	Strategy consultant
S12	Food producer association	Vice president
S13	Agrifood services association	Agricultural practice consultant
S14	Certification company	Manager
S15	Cultural association	Project manager
S16	Consumer association	Manager
S17	NGO sustainable agriculture	Researcher and founder
S18	Regional authority	Head of department
S19	Regional authority	Head of department
S20	Food producer	Food quality manager
	Norway	
S21	National authority	Senior advisor
S22	Consumer association	Technical director
S23	NGO Norwegian food	Manager
S24	Food producer association	Director for analysis and policy
S25	Food producer association	Analysis manager
S26	Food producer association	Manager for strategy and development
S27	Food producer association	Communication manager
S28	Certification authority	Team leader
S29	Environmental organisation	Advisor in food production and agriculture
S30	NGO sustainable food	Project coordinator
	Romania	
S31	National authority	Head of department
S32	Food producer association	President
S33	Research organisation in the environmental field	Researcher
S34	Health association	Financial assistant
S35	National authority	Head of department
S36	Association of agrifood producers	President
S37	National agency	Legal advisor
S38	Consumer association	President
S39	National authority	Head of department
S40	Environmental association	President

## Data Availability

The data presented in this study are available in English language in Table A2 and Table A3 in Appendix A.

## Appendix A

**Table A1:** Supertext template for critical frame analysis.

Part 1: General information about the text Number/code/Title: Full title: Country/Place: Regulation X (out of 10) Issue: Release date: Status of document:
Part 2: Specific information on the text Voice: Voice(s) speaking: Perspective: References: Diagnosis: Problem: Why it is seen as a problem(s): Actors of the problem (who is responsible for making the problem, who is responsible for solving the problem): Causality (cause of the problem(s)): Dimensions of the issue (social categories and behaviours): Intersectionality (other issues related to the main one): Mechanisms (resources/norms used in the text): Form (persuasion techniques/metaphors/contrasts used in the text): Location (what level the text refers to): Normativity (what is normal if that is the problem): Legitimisation of non-problem(s) (what should or should not be legitimised): Prognosis: Proposed solution: Priority goals and their order: How to achieve the goals (strategy/means/instruments): Social categories that should act and the desired behaviour: Who should [not] do what: Form (argumentation/style/persuasion techniques/metaphors): Where the solution should be applied (national/regional level, local projects, NGOs, etc.): Normativity: What is seen as good? What is seen as bad? Balance: Balance in the analysed text (correlation between diagnosis and prognosis): Frictions or contradictions within the elements of the text:
Part 3: Further analysis—Comments (1) Identification and construction of issue frames (2) How document frames combine into issue frames (3) Are there any metaframes by finding common normative frames belonging to different policy issues?

Source: Adapted from Verloo and Lombardo (2007) and Dombos et al. (2012).

**Table A2:** Relevant sustainability dimensions, drivers, barriers, and solutions related to SFSs in the national legislative/policy documents according to the critical frame analysis.

	Food Sustainability Dimensions	Drivers of and Barriers to SFSs	Solutions for SFSs
Germany			
D1	Climate change; biodiversity; resource management and other environmental issues	Consumption of products and services significantly influences not only the economic and social situation but also the state of the environment; household consumption with great potential to reduce the environmental impact	National strategies for sustainable development including debate within society; education, consumer information; environmental and social labels; ecodesign; sustainable public procurement; research on sustainable consumption; social innovations, monitoring sustainable consumption; adopt a circular economy strategy and increase research, innovation, and training on green economy; attention to marginal areas; additional funds for agrifood business and the implementation of environmental related initiatives; keeping vital rural areas
D2	Labelling of organic products	Information on the label; information about the products; role of and the correct use of certification; certification	Correct and transparent labelling system; informing consumers; supporting certified products; constant evaluation of organic certificated food products; safeguarding environmentally friendly production
D3	Organic food production	Organic farming is resource-efficient and environmentally friendly in line with the principle of sustainability; demand for organic farm products has been increasing, and this demand can only partly be satisfied at this point in time	Expansion of organic farming practices; five major action points (coherent legal framework, improve ease of access to organic farming, making use of existing and expanding demand potential, improve performance of ecological agricultural systems, reward environmental services) are suggested and described
D4	Food waste, as a waste of resources, to be avoided from economic, ecological, and ethical perspectives	Waste of food by companies that could consider passing on food items to social institutions but do not do so because of legal concerns	Reducing food waste and providing food for people in need; setting up a system for donating food products close to the expiration date to the endangered social categories; providing clear and easy-to-understand information for donors and recipients of food items
D5	Greenhouse gas; processing and consumption of food, social impacts through choices of food	Climate change, health, and the global population because our food consumption is increasing rather than decreasing; there is need to act in this area and help guide a shift towards more sustainable food consumption	Guidance from specific institutions for individual consumers with regard to choosing sustainable food consumption; controls from supervisory authorities in the case of food production and animal welfare
D6	Environmental protection; soil conversation; protection of species; water pollution; animal welfare; reduce of negative impact of food production by using organic farming	Limited number of organic agricultural enterprises, although organic farming methods are more sustainable	Guiding society towards more sustainable food production; five main actions points with 24 concepts are defined, addressed to the government and producers
D7	Labelling of organic products; use of national and EU organic label	Voluntary national organic label in combination with the EU organic label	Use of national organic labelling whenever possible and appropriate
D8	Genetic modification of food and feed	The underlying cause is fear of GM foods and feeds, and related health concerns	Limiting the use of genetically modified food; informing consumers and producers
D9	General principles of sustainability, including all 17 SDGs	Difficulty following all indicators and activities that are required to achieve SDGs	Strengthening policy coherence and the inclusion of social actors; improving the work of institutions, federal and state government cooperation; prioritising the German sustainability strategy by departments, sustainability program for public actors and facilities; organic agriculture should represent 25% of total agriculture in Germany by 2025
D10	Sustainable food; labelling; government initiatives related to sustainable food consumption; CO_2_ reduction	Changing habits in daily life related to food shopping; easy and time-saving food shopping	Guidelines addressed to German consumers to guide them towards more sustainable food choices; priority goal is the reduction of CO_2_ emissions and other impacts on the environment caused by our food choices
Italy			
D11	Environmental protection; use of natural resources	Protection of water resources; correct agricultural practices; management of waste; favour interventions for environmental protection; territorial and regional governance	Environmental standards; environmental and social considerations in public decision making; measures related to agrifood activities (e.g., protection water resources, agrifood heritage, and landscape); adaptation of territorial plans and regional programs to the goals of resource protections
D12	Green economy; use of natural resources; environmental protection	Competitiveness of national production; circular economy; role of public administrations in stimulating ‘green’ behaviours; protection of flora and fauna; recycling; production of energy; protection of water and soil resources	Label ‘Made Green in Italy’; Green Community and National Strategy of Green Community; environmental certification for environmental provisions; payment for ecosystem services; concerted actions (e.g., Committee for the Natural Capital); more awareness on sustainable mobility and city
D13	Environmental protection; use of natural resources; social issues; countries’ vulnerabilities	Natural resource preservation; attention to marginal, abandoned, and rural areas; attention to vulnerable farmers and fishers; enhancement of agrifood sector; consumer protection; issue of single-use plastics; renewable energy; citizens’ awareness	Additional funds for agrifood businesses; environment-related initiatives; fight against food frauds and misleading information; particular attention to marginal areas; food waste and donations
D14	Biodiversity preservation and enhancement; promotion of biodiversity for agricultural and food purposes; consideration of social aspects	Risk of genetic erosion and extinction; role of farmers; citizens’ awareness; product differentiation and typicality; preservation of rural areas; keeping vital rural and marginal areas	Biodiversity community; initiatives for biodiversity: national register, national network, plan and guidelines, central committee of management and monitoring, educational and informative activity, support for research
D15	Competitiveness of agricultural sector; keeping vital rural areas; supporting marginal areas	Role of vital farming; diversification of income sources for farmers; multi-functionality of agriculture; territorial cooperation; local resources and local products; preservation of rural areas	Label ‘Mountain Product’; traceability along all stages of the food supply chain; food and rural districts; correct and transparent labelling system; inter-ministry committee on food safety; concerted actions (e.g., agrifood discussion table); particular attention to marginal areas
D16	Green economy; environmental protection; energy efficiency; social needs; public health	Role of public administration contracts in stimulating environmental and social responsibility among businesses and citizens	Environmental and social considerations in public decision making; green public procurement
D17	Green economy; sustainable development; supporting marginal areas; food safety	Transition to a green economy; relevance of green public procurement; competitiveness of national companies in international markets; traditional agrifood products; waste management; scarce attention on ecological issues by previous national policy makers	Environmental and social considerations in public decision making; green public procurement; circular economy strategy; research, innovation and training on green economy; promotion of Made in Italy; correct and transparent labelling system; integration of rural development measures with interventions of general interests (e.g., landscape protection)
D18	Consumer/citizen protection; correct information	Respect for labelling requirements; transparency in origin, ingredients and other product information; informed choices and effects on health; fair labelling practices; ensuring truthful information	Correct and transparent labelling system; fight against food frauds and misleading information; punishment of deviations from mandatory requirements
D19	Improvement of public health	Health promotion; reduction of health inequalities; governance of national health system; health awareness; role of food choice and eating behaviour on health	Capacity-building initiatives to support policy making about health; fight against food frauds and misleading information; education campaigns for citizens
D20	Sustainable growth; relevance of research for agricultural sector	National strategy for research and innovation of agricultural sector; increase in productivity, profitability and efficiency; sustainability indirectly considered in different aspects (e.g., use of inputs, new technologies of production, energy, and non-food purposes); role of young farmers	Research and innovation in: sustainable increase in productivity, profitability, and resource efficiency in agroecosystems; climate change, biodiversity, soil functionality, and other ecological and social services of agriculture; coordination and integration supply chain processes and strengthening the role of agriculture; quality, typicality and food safety, and healthy lifestyles; sustainable use of biological resources for energy and industrial purposes; development and reorganisation of the system of knowledge for the agricultural, food, and forestry sector
Norway			
D21	Sustainable growth for aquaculture both in economic (competitiveness, capacity increase) and environmental (adaptation to environmental changes) terms	Aquaculture industry ‘reached its limits’; new permits and rules are needed to ensure further growth; need for new guidelines for maximum permitted biomass, when and how to seek increased capacity	Increasing the aquaculture industry’s production capacity in areas with little or moderate environmental impact, while reducing capacity in areas with unacceptable influence; promoting the aquaculture industry’s profitability and competitiveness within the framework of environmentally sustainable development
D22	Organic production; animal welfare; best environmental practice; biodiversity; labelling of organic agricultural products, aquaculture products, food, and feed	Understanding of what is considered organic and not; labelling organic products; benefits of organic labels; misuse of organic labels; regulations for what can be categorised as organic	Rules and clarifications for applying organic labels; regulations on organic production and agriculture
D23	Development of agriculture-related industries; increasing settlement in rural areas; rural development	Renewal of agriculture-based industry; overpopulation of urban areas and underpopulation of rural areas; developing rural areas; promoting organic production and locally grown food	Collaboration in the regions, and between regional and national levels, to create profitable jobs and contribute to employment throughout the country; agriculture-related business development as a tool for achieving district policy goals
D24	Food information; food safety; health; consumer awareness; avoiding misleading information	Lack of information for consumers about nutritional content, production method, origin, packaging, and pesticides; misleading actions and omission of important information; consumer knowledge about health, economic, environmental, social, and ethical issues	Providing rules of food information for consumers; regulation to ensure quality and consumer considerations along the entire production chain, and to safeguard environmentally friendly production
D25	Biotechnology; genetically modified organisms; uncertain adverse effects; social responsibility; ethics; animal welfare; human health	Lack of information about the outcomes of gene modification on animals, people, and the environment; ensuring animal welfare and ethical and sustainable principles when using genetic engineering	Restrictive policy limiting the use of genetically modified food with a set of rules that safeguards social responsibility, animal welfare, sustainability, and ethics
D26	Food safety; human health; animal and plant health	Risk to human health if the actors do not run their ventures compliant with the law; economic and labour-saving methods benefiting the actor but threatening human health	A set of regulations (e.g., about hygiene activities); controls from supervisory authorities
D27	Food safety; animal and plant welfare; human health; authorisation; regulations on pesticides; environmentally friendly production	Problems caused by pests, fungi, and weeds damaging living plants, plant parts, and seeds; supervision of plant and food safety, plant health, animal health, and animal welfare, and health, quality, and consumer concerns throughout the food production chain	Updated and more relevant regulations on the use of pesticides to safeguard plant welfare and ensure food safety
D28	Animal welfare; animal treatment; animal owners’ responsibility	Varying attitudes towards animals’ care, welfare, and living conditions; treating animals in an irresponsible and unethical manner	Regulations obliging to help if one finds an animal that is sick, injured, or helpless or has reason to believe that animals are subjected to abuse; strict rules regarding labelling, tracking, transport, killing, trials, living environment, breeding, and animal care; control of farms from supervisory authorities
D29	Animal welfare; animal needs; animal living conditions; animal care	Varying attitudes towards animals’ care, welfare and living conditions; treating animals in an irresponsible and unethical manner; poor animal health and diseases due to bad living conditions	Regulations to ensure good living conditions for animals; regulations obliging to notify the authorities if one has a reason to believe that animals are subjected to abuse; control of farms from supervisory authorities
D30	Animal welfare; animal needs; animal living conditions; animal care	Varying attitudes towards animals’ care, welfare, and living conditions; treating animals in an irresponsible and unethical manner; poor animal health and diseases due to bad living conditions	Regulations to ensure good living conditions for animals; regulations obliging to notify the authorities if one has a reason to believe that animals are subjected to abuse; control of farms from supervisory authorities
Romania			
D31	Labelling of organic products; correct information; consumer/citizen protection	Information on the label; information about the provenance of products; role and correct use of certification in influencing consumers (which could be used to mislead them); recertification	Re-evaluation process for organic products; stop selling products with the old label
D32	Environmental protection, quality of the environment, animal health, human health; food safety, correct information for farmers	Controlling the selling and use of agricultural pesticides; role of producers and retailers of pesticides in influencing farmers’ use of pesticides; water, air, and soil pollution from pesticides; complying with the EU regulation	Engagement to set up a National Action Plan to reduce the use of harmful pesticides in agriculture, which includes the obligation of sellers to advise farmers of the use of less harmful and even organic products; also, it includes restraining the use of pesticides, which spread in the air
D33	Conversion to organic agriculture, sustainable agriculture; labelling of organic products; food production	Clear definitions of terms related to organic production; methodology of organic production; simpler guidelines for the labelling of organic food products; favouring the conversion to sustainable agriculture	Solution is presented as a series of steps that need to be taken to become organic producers; a specific type of label is offered by the certification authority (National Authority for Organic Products) to inform consumers regarding the origins of the product; food operators are called out to apply for the conversion of their farms or processing factories into organic ones, and to certify their products
D34	Organic production in countries outside the EU; organic agriculture; European standards, quality standards; organic certification; informed consumers	Different understandings of organic production by the countries outside the EU; quality of imported non-EU products	Authorities must verify each lot of imported agrifood products regarding the documentation and actual quality of merchandise through a set of rapid analyses at the border so that the documents are correlated with the merchandise
D35	Human health; animal welfare; monitoring food production; risk monitoring; food safety	Rapid changes in the food production system and the logistic systems; informing consumers about possible hazards in a short time; clear responsibilities of food producers and food retailers in the area of food safety; monitoring of risks and hazards in the entire food production system	National Sanitary Veterinary and Food Safety Authority should constantly monitor all food producers and retailers, who should adhere to the hazard monitoring system and to the rapid alert system; there is a need to set up a national structure responsible for regulating, analysing, and monitoring all possible situations in the matter of food safety for both humans and animals
D36	Food waste; food donation; recycling; reuse (environmental protection); consumer education and awareness; social endangered categories	Increased food waste; volunteer actions of reducing food waste by large food operators; consumer education and awareness of food waste issues generated by high consumption	Participation of or setting up by the economic operators of information campaigns on food reduction for end consumers; a set of measures that could extend the life of a product, even if it has expired, such as selling the products at a low price or donating the product to a social organisation
D37	Eco-conditionality and cross-compliance regulations (environmental protection); animal rights; human health; funding in agriculture	Current agricultural system is harmful for the environment, animals and human health; funding farmers’ activities only if the norms of eco-conditionality are complied with; compliance with eco-conditionality and cross-compliance regulations	Farmers must limit the fertilisers and pesticides used in agriculture; converting farms for agriculture with more respect for the environment and living beings; follow European eco-conditionality and cross-compliance norms; authorities should constantly verify farmers’ compliance with the regulations
D38	Organic agriculture label; components/composition of food products	Missing information on the label about the provenance of organic food products	The proportion of organic ingredients should be mentioned on the label of organic food to properly inform consumers about the use of the ‘ae’ label on food products
D39	Traditional food products; certification, labelling rules; producer and consumer protection	Labelling rules and use for traditional food products; promoting traditional food products; counterfeit traditional food and unfair competition in the market	Authorities must closely verify compliance with traditional product criteria and conditions; authorities should constantly monitor the impact of the regulations of traditional food producers; producers must respect the conditions and criteria when asking for traditional product labels and after receiving them
D40	Sustainable development; environmental protection; health issues of all living beings	Unsustainable development; historical and geographical aspects of a country; inequalities from economic, social and environmental perspectives	Set up a comprehensive agenda for Romania, considering the 17 SDGs, with clear targets, for contributing to the sustainable development of the world, as part of it

**Table A3:** Relevant sustainability dimensions, drivers, barriers, and solutions related to SFSs highlighted by interviewed stakeholders according to qualitative content analysis.

	Food Sustainability Dimensions	Drivers of and Barriers to SFSs	Solutions for SFSs
Germany			
S1	Climate-friendly diet; environmentally friendly food; resource-efficient production; certification	Habits to buy and cook what has always been bought and cooked; vegetarian dishes are less appealing in big cantinas than meat dishes; lack of knowledge; food is a sensitive and individual topic for people, people do not like to be told what to eat	Increase attractiveness of vegetarian dishes, meat-reduced dishes, vegan dishes also in public cantinas; visualisation of information at the point of sale for greater appearance of sustainability; implement second price tag that includes environmental costs; apps to contribute to knowledge about sustainable food choices/organic farming; CO_2_ tax or adjustments of the VAT for food production
S2	Cradle to cradle system (production and consumption without waste); increasing the share in consumption of organic and regionally produced food	No official definition for ‘regional’ that is legally binding; sustainable food items required many assumptions and definitions; limited transparency; policy must pay attention to economic viability	Education of producers and consumers; transparency to reduce complexity; awareness of sustainable food through nudging, campaigns, education, and nutritional labelling
S3	Organic farming, including the ecological and social system	Availability and ease of accessibility of sustainable food to consumers; young parents start buying organic food because of baby food; price	Communication; transparency; awareness of the consumer; presence of sustainable food choices in public cantinas
S4	Planetary boundaries related to the food system (i.e., basic agriculture, water, soil, biodiversity, and ethical issues)	Abundance in the food sector by cheaply produced non-sustainable food; affordability (price and accessibility); mistrust through fake news, fake food; sustainable food is an implementation problem; habits and tradition of food choice; easier adaptation of younger generations; development in some groups to be ashamed of making unsustainable choices	Measures needed to achieve a circular economy that keeps the planetary limits to maintain productive systems that are stable; climate adaptability; advertisement and marketing; reaching young people by attention economy; framework set by politics; organic should be the standard, conventional should be labelled
S5	Subsisting on the strictest possible state regulations, namely the EU ordinance or other directives on organic agriculture; animal welfare	Increasing demand for sustainable/organic food choices in the last 20 years; animal welfare, regionality, and healthiness; digital tools as chance for the younger generation; lack of availability in out-of-home catering; strong conventional agricultural lobby	Increase in research funding for organic farming; accompanying campaigns in the field of organic in the out-of-home catering; education; facing consumers with their food choice habits; promotion supported by politics
S6	No more relying on the three-pillar model, but focused on the question how we can feed ourselves sustainably so we can keep the borders of the Earth and maybe even regenerate the planetary systems	Question of time, of the right information and of the right conditions and framework; producing food under unfair competitive conditions, unfair to the environment and to people; lack of experience and knowledge of transformation	Social-ecological tax reform (price of using natural resources needs to be reflected in the prices of food); experiential education, away from the theory, but showing people how food is produced, how recycling works
S7	Human consumption that makes the lifestyle transferable and scalable; in terms of the environmental factor, minimising the environmental footprint; in terms of social impacts, enhancing the positive impact on global supply chains; in economic terms, allowing the individual wallet to play a role, to not make this an upper-class theme	Affordability (price and accessibility); lack of a common approach to sustainability along the supply chain	Education (e.g., nudging); transparency; public procurement and cantinas to influence people’s food choices; real costs approach, real prices; credibility of labels
S8	Sustainable food system is basically a circular economy; no waste production of food and packaging; pricing system that reflects costs and benefits; cradle to cradle system	Complicated and heavy communication; communicate topics around sustainability too much in terms of renunciation, abstinence from flying, abandonment of mangoes, abandonment of meat	Communicate in an understandable and uncomplicated way; true cost approach; experience-oriented education; political acting through procurement
S9	Three pillars principle of sustainability; as far as the environment is concerned, it is clear that a system must be designed in such a way that it does not overburden and excessively affect resources; social principles are not violated in the complete value chain	Costs and lack of knowledge; thoughtlessness of consumers; main drivers are consumers and society; lack of belief in organic labelling	Guerrilla marketing; introduce CO_2_ taxation; experience-based education in schools for the younger generation; raise awareness in public and use responsible actors; apps or other digital tools only for already-aware consumers; introduce the system of responsibility in the industry
S10	Three pillars principle of sustainability with a focus on ecological and social aspects; health aspect of food consumption	Costs and lack of knowledge; age of the consumer (younger generations are more flexible); strong conventional agricultural lobby	Offer sustainable products without huge marketing communication; more restrictive regulations for animal welfare by politics; move all standards towards organic farming; placement of organic products in supermarkets
Italy			
S11	Organisational change in the supply chain; new relationship between producers and consumers; knowledge, know-how, competence, and culture; biodiversity in genetics, environment, and culture; more than organic food; not only product innovation; no more an option	Affordability (price and accessibility); too much information and fake news; consumers’ propensity towards sustainability; role of public institutions; role of the EU	National food safety agency; information quality from journalists and scientific communicators; building sustainable food supply chains involving all actors (consumers, retailers, manufacturers, farmers, public institutions); product-specific cooperation for SFSs; back to tradition, territorial specificities, culinary culture; more agrifood supply chain policies from EU
S12	Fair income for workers and producers; application of non-polluting practices in the supply chain; organic food (the only one regulated by law and certified); irreversible process; biodiversity and citizens’ culture on biodiversity	Daily choice depending on consumer culture, knowledge and attitudes on wellbeing, health, and environment; price and values associated with sustainable food; large retailers’ policies	Regulations and policies to facilitate sustainable processes; promote organic food at schools and public administrations; education at school; certification, controls, and monitoring; accessibility to make the choice of sustainable food easier; traceability through technology; more free and independent research; generational change in agriculture; monitoring information food labels; shared objectives and cooperation along the supply chain
S13	High-quality and healthy products; respect for ethics and moral values; fair prices reflecting production efforts; reassurance to consumers about producers’ commitment; respecting the law or being proactive with the law	‘Intrinsic dualism’ in consumer choice (high-processed food v. natural food); price; consumers’ awareness and education; information (fake news and distorted information); role of public institutions; role of technology; large retailers’ policies; lack of a common approach to sustainability along the supply chain	Regulations and policies facilitating sustainable processes; more information at the point of sale; support producers adopting sustainable practices; more education for young generations; more investment in scientific research, new technologies, innovation implementation, and infrastructures; management of food waste and food packaging; promote circular economy
S14	Three environmental, social, and economic pillars; nutrition; related to the product, business and territory; attribute generating differentiation; fair and safe working conditions; fair income to farmers and along the supply chain; circular economy and waste management	Lack of common sustainability models, approaches, and standards; gaps in communication and confusion among consumers; feasible, verifiable, and certifiable standards; price; role of technology; role of public institutions; not only for large businesses; involvement along the food supply chain	Shared definition of standards (requirements and measures); considering food supply chain peculiarities; support ‘Made in Italy’ in terms of sustainability; reinforce Protected Designations of Origin through sustainability; more attention to animal welfare; more involvement of large retailers; education at school
S15	Three environmental, social, and economic pillars along the supply chain; organic production; sustainable packaging; local transportation and distribution; fair and safe working conditions; circular economy and waste management; sufficiency criterion	Price and environmental cost; interest of young generations; lack of common sustainability models and approaches; long-term planning; role of technology; role of public institutions; education; role of EU	Build a sense of community and participatory process; sustainability as an experience; partnership between producers and consumers; solidarity purchasing groups; information technology (e.g., talking labels, block-chain, apps); support producers adopting sustainable practices, and inter-professional networks
S16	Production and consumption model inspired by low environmental impacts; technological–scientific revolution; consumer purchase model based on awareness about green products (i.e., respecting the environment, reducing waste, promoting reuse and recycle)	Interest of young generations; misleading claims and information; role of technology; role of public institutions; large retailers’ policies—the power of consumer choice	Support producers adopting sustainable practices; more investment in R&D; promotion of fair conditions and skill improvement for workers in food supply chains; information technology (e.g., talking labels, block-chain, apps); more education; new marketing strategies increasing trust in brands and certifications, higher food quality, reducing portion size and correct advertising
S17	Change of habits to respect production seasonality; nutrition; biodiversity and localness; promotion of natural and cultural heritage	Interest of young generations; information and environmental costs; lack of media attention; poor nutrition and health issues; misleading claims and advertising; large retailers’ policies; education; role of public institutions	Agroecology; more attention to nutrition; education at school; promotion of agrifood heritage; promotion of local consumption and waste reduction; information technology (e.g., talking labels, block-chain, apps); more public measures for biodiversity and reducing desertification
S18	Three environmental, social and economic pillars; certification; nutrition; natural resource protection today and for the future	Lack of common sustainability models, approaches and standards; role of public institutions in measuring sustainability; price and willingness to pay; information and packaging labelling; education; producers vs. citizens in the use of territorial resources; waste management; role of technology; large retailers’ policies; business marketing strategies	Less bureaucracy for producers; shared definition of standards (requirements and measures); considering food supply chain peculiarities; promotion of inter-professional networks and skill development; supply chain certification; support producers adopting sustainable practices, start-up, investment in R&D; information technology (e.g., talking labels, block-chain, apps); education at school; green public procurement; make sustainability a ‘lifestyle’
S19	Three environmental, social, and economic pillars—certification; food security and safety	Accessibility; transparent information in stores; lack of common sustainability models, approaches and standards; business marketing strategies; large retailers’ policies; education; information; role of big data; role of the EU	Promotion of inter-professional networks and skill development; shared definition of standards (requirements and measures); definition of a minimum level of sustainability; public supervision of production costs and market prices of sustainable food
S20	Three environmental, social, and economic pillars; certification; nutrition and health; high quality; ethics; biodiversity and localness; promotion of natural and cultural heritage	Lack of common sustainability models, approaches and standards; misleading advertising; education; correct information; lack of norms regulating high-quality product characteristics; poor nutrition and health issues; high-processed food and nutritional depletion; correct use of technology; interest of young generations and ‘intrinsic dualism’; role of the EU	Make sustainability a ‘lifestyle’ and promote healthy products; more public measures for biodiversity and food culture protection; more attention to nutritional standards of sustainability and business environmental impacts; role of public institutions in sustainability certifications; investment in transportation and logistics; education and information; information technology (e.g., talking labels, block-chain, apps); support producers adopting sustainable practices; reduction of portion size
Norway			
S21	Social and cultural dimensions of sustainability (e.g., affordability of good and healthy food); having an understanding of how all humans everywhere should be able to produce food that gives a complete diet	Need for a broader understanding of what sustainable food is, especially among young people; lack of knowledge about sustainable food; food sustainability is a complex multidimensional concept	Importance of influencers; increase consumer engagement for sustainable food; make sustainable choices easier and more understandable (labelling, guidelines); education about sustainable food in schools; encourage consumers to do simple things like sorting waste and eating varied and locally produced food
S22	Living in a way that allows us to leave behind the same resources and living conditions that we ourselves now have to our younger generations and descendants; eating food that creates less CO_2_ emissions, but still gives us the nutrition we need	Lack of knowledge about environmental changes, sustainability, nutrition, and healthy alternatives; habits and traditions, to a large degree, shape people’s diets and create resistance to change; lack of availability and affordability; food waste concept; conflicting messages about what sustainable food is	Make sustainable choices as simple and available as possible (e.g., providing vegetarian and vegan options at every restaurant); provide knowledge about sustainable food; large distributors (e.g., supermarkets) need to provide more sustainable food choices; redefine what food waste is—we need to define all loss of food as food waste; have a more wholesome understanding and approach towards sustainable food with a variety of different actors using one voice
S23	Complex concept with many different, often conflicting, angles; question about more than emissions and pollution—it is also, for example, about working and living conditions for employees at tomato farms	Conflicting information about food sustainability; high prices for sustainable food, especially for young people; availability of sustainable food	Storytelling as a powerful tool to attract consumers; supermarket chains can provide more sustainable food options; clear, easy-to-understand and transparent requirements for what sustainable food is, strategy and funding for promoting sustainable food; encourage simple actions (e.g., reducing food waste, decreasing weekly meat consumption)
S24	Compliance with UN’s SDGs; includes sustainable economy, removal of both hunger and obesity, environmental impact, and greenhouse gas emissions	Conflicting professional recommendations on food sustainability; need to provide the right information to consumers; food waste and plastic waste	Use media to increase engagement around sustainable food; label sustainable food products; provide information to consumers; government needs to regulate, coordinate, and facilitate knowledge and coordinate district politics, agricultural politics, and industrial politics; use digital technologies to capture data throughout the whole value chain; provide official dietary advice on sustainable food; reduce food waste and plastic waste
S25	Climate and CO_2_ emissions (e.g., meat production produces a lot of CO_2_); different views on sustainability (e.g., meat producers in Norway believe that meat production is sustainable due to the Norwegian landscape and climate conditions)	‘Zero waste’ and climate campaign often come across as too hysterical for many people; availability of sustainable food; affordability of sustainable food; lack of knowledge about sustainable food	Increase consumption of fruit and vegetables; power of communication (e.g., through social media); teach consumers about sustainability; provide fresh and fast sustainable food (e.g., pre-cut fruit and vegetables at every supermarket and kiosk); school fruit project; reduce food waste by providing special offers for food that is about to expire; label sustainable food; have a comprehensive approach towards food sustainability taking into consideration different sustainability aspects; provide local food; provide knowledge about sustainable food (e.g., making sustainability a bigger part of the school curriculum); make sustainable alternatives more attractive (e.g., by communicating their positive effects)
S26	Multidimensional concept; it is not only about the environmental aspect, but just as much about the use of resources, nutrient-dense products (e.g., if you cut out meat, you will probably need product a, b, and c to substitute those nutrients), economy, and ensuring food safety	Food sustainability concept feels vague and confusing; food waste; food sustainability feels too overwhelming; need to adjust the definition of food sustainability to local conditions	Use influencers such as bloggers and YouTubers; encourage consumers to do simple things to enhance sustainability (e.g., weekly meat-free day); all actors in the food system (e.g., farmers, manufacturers, stores, and distribution) should take responsibility; take local circumstances into account when discussing food sustainability; a balanced approach towards food sustainability; technology to increase food sustainability; decrease food waste (buy less, smaller portions)
S27	Importance of choosing locally produced food; using as many parts of the animal as possible; doing one’s best to reduce food waste; supporting local producers	Affordability of sustainable food; availability of sustainable food; strict regulatory framework that, to a large degree, prevents using leftover foods; media focuses mostly on cheap food, not sustainable food; Norwegian producers lack marketing skills to promote their products	Distributors can influence consumers (e.g., through product placement); Norwegian producers need to improve their marketing of local food products; media needs to focus on food sustainability; reduce fees and taxes for Norwegian food; Norwegian food must be served at all public events; reduce meat consumption and eat better, ethically produced meat, eat more local products; further research to make food more sustainable; develop new technologies to further support sustainable production of food
S28	Complex concept that can mean both everything and nothing; a kind of attitude towards how you orient yourself in society, that you look at the bigger picture when you make choices and think not only about yourself	Availability of sustainable food; transporting food over large distances (e.g., by plane); food waste because what the population acknowledges as food is limited (e.g., not eating all parts of the animal); lack of knowledge about sustainable food and sustainable agriculture	Use role models and positive examples (e.g., influencers and famous people); producers can make sustainable food more attractive (e.g., through packaging design); reduce food transport; remove the VAT on ecological food and on fruit and vegetables; further develop organic agriculture; increase knowledge of biological processes, ecosystems, and agriculture in general; reduce food waste (e.g., eat the whole animal, sell expired food at lower prices, plan for grocery shopping); make sustainability a bigger part of the school curriculum
S29	Food produced with regard to the climate and common justice at all production stages, and based on local resources; food produced to the largest possible degree locally in Norway	Lack of knowledge about sustainable food; availability of information about sustainable food; availability of local food; involvement of large grocery chains; affordability of sustainable food	Food producers need to be honest and transparent with their information on how food is made; provide information and knowledge about food sustainability to consumers; implement sustainability as part of the school curriculum; big grocery chain stores should explicitly explain the consequences of choosing or not choosing sustainable food; promote local foods using good stories; open alternative channels for the distribution of local food (e.g., ‘Reko-ringer’); more laws and regulations regarding animal feed and animal products (e.g., labelling); dedicate more agricultural area to plant-based protein, such as peas and legumes; regulate price levels, so that sustainable food options do not become too expensive; launch support schemes for farmers selling directly to consumers
S30	Change from a meat-based to a more plant-based diet; in every product category, find the alternatives that score high on water and land sustainability and CO_2_ emissions in general, through the whole value chain	Lack of knowledge and information about sustainable food; availability of sustainable food; conflicting messages between what is healthy and what is sustainable; affordability of sustainable food; food sustainability seems to be too complicated; food waste	Increase knowledge of sustainable food among consumers; increase availability of sustainable food in supermarkets and restaurants; government needs to make more requirements and guidelines to make it easier for companies and the public to live more sustainably; provide better prices, placement, and product design for sustainable food; labelling of sustainable food; more control and transparency on how food is produced within the EU; more research-based approaches to what actually works and what does not work for promoting sustainable food; reduce food waste (e.g., smaller portions)
Romania			
S31	Social benefits; healthy lifestyle; environmental protection; ethics; environmental respect; local products	Food security; food waste; climate change; natural resources depletion; availability of natural resources; degree of specialisation of producers; implementation of environmentally friendly production techniques; availability of sustainable food; information on producers of sustainable food; degree of logistics technology; short food chain; storage, transport, and traceability; connecting to RNDR (national rural development network); consumption patterns; reluctance to change food habits and to try new food products; information; income; geography (urban/rural); quality schemes and compensatory payments; fiscal, legislative, and coercive measures; national awareness campaigns on sustainable food	Develop a national rural development network (a project or organisation) that gathers producers, consumers, public local units to put them in contact to shorten the link between them and create new partnerships in the field of rural development, while promoting sustainable development
S32	Food access of humans and other organisms; without impacting resources for future generations; food waste reduction; ethics; preservation of local heritage; synergy between local actors	Education and information; consumerism policy; prices as influencing factors of demand; public policies for sustainable food system development; knowledge of policy makers in terms of the quality system of food products; financing measures on quality and promotion of food	Create products of certified quality; implement quality schemes; stronger marketing campaigns; stimulate the desire of consumers for sustainable food purchases; higher taxes for unsustainable producers, which further stimulates consumers; introduce a national program that stimulates food exports; education of consumers, awareness campaigns; introduce a sustainability criterion in public procurement and project assessment for funding; create a national framework, developing strategies for Romanian food; the role of government in fostering the development of a sustainable food system could be achieved by creating a legislative framework and information campaigns on sustainable food for consumers
S33	Local economy; economically viable; environmental and health protection; balance	Information regarding environmental imbalances and effects generated on communities; health awareness; consumers’ preferences and education from young ages; market competitiveness of the traditional household; intensive agricultural practices; unfair competition between local and foreign products; focus should be on processed food exports and not on agricultural exports; length of the supply chain; acquisition processes; current promoted models of agriculture; market promotion	Create the direction and provide the necessary financial measures (main role of the state); elaborate technical studies and analyses of the current situation and then implement promotion campaigns to educate consumers
S34	Resource conservation and protection for future generation; available resources; environmental protection; nutrition; quality of life; prosperity; economic growth	Information campaigns; knowledge of the meaning of sustainable products; consolidation of legislative framework; access to healthy food; prices of sustainable food (fair); development and restrictions of production processes; promotion of innovation in terms of producing goods with both a minimal impact on the environment and comfort for users; marketing campaigns for sustainable products and consumers’ education; diversification of sustainable food	Label sustainable products, add more information on the label regarding the content of the product; develop green shops; develop the recycling infrastructure in shopping areas; develop the legislative framework; more involvement of public authorities in developing the SFS; increase the availability (production, affordability) of sustainable food for disadvantaged social categories
S35	Biodiversity protection; good level of life and a long one; food choice; information; balance between nutritious eating, environmental dimensions and the human system for heading towards the ‘blue area’	Economic interest of stakeholders; demand; consumers’ affordability of the sustainable food; financial benefits, economic incentives, administrative facilities, and market organisation facilities; information held by consumers; education and information; higher living standard	Develop a strong and coherent policy; organise events to raise awareness in various social categories; support producers by granting benefits; develop various associative forms; stimulate the demand for sustainable food
S36	Food availability; acknowledging planetary boundaries; giving back to Earth; not harming the planet; environmental protection; balance	Lack of knowledge (insufficient reading); lack of role models and opinion leaders; education and information; chaos on the internet; distribution of income; ‘business owner’ mentality (everyone should have a business, which generates over-competition); production costs; financial opportunities for clusters, research; stringent European market policies compared with other countries around the world; consumers’ tastes and habits; prices; disagreements between the manufacturers of different products; excesses of consumption; political interests; technological innovation and development; digitisation, adaptation to change	Change the financing paradigm by financing real sustainability and reducing taxes for sustainable practices; create a new business model; finance and develop digitalisation; finance and develop the research field; true education; take responsibility for communication on SFSs by opinion leaders and role models; create digital standards and digital platforms; workforce reconversion; reassess of the current evaluation grid used for project evaluation to be funded by considering all aspects of sustainability; create a strategy for SFSs; develop a global system of analysis, collection, algorithm, prediction, and preventive measures of risks in the food chain
S37	Environment’s gain; consumers’ gain; good quality; thinking of future generations; innovation and technology	Lack of information; lack of the desire to be informed; non-involvement of decision makers; power of example; education; information; mass media; innovation; financial incentives; presence of environmentally friendly distribution and technologies	Raise awareness about the meaning and benefits of sustainable food; develop educational programs
S38	Organic food; traditional food; tackling the natural resources depletion; environmental protection	Lack of resource allocation to support producers; frequent controls that disrupt activities; lack of opportunities to support sustainable producers; purchasing power; lack of knowledge; role of the state; awareness campaigns; accessible prices	Promote national information and education campaigns over several years; provide financial incentives to sustainable food producers; find new markets to sell sustainable products
S39	Environmental protection; health; ethics; balance and measure in all; local products; local suppliers; products with no content of chemicals or genetically modified ingredients; waste reduction; reuse and recycle waste; elimination of useless imports	Availability (access in terms of quantity and quality and affordability); education; habits; prices; producers’ stimulus; faster adaptation to change of younger generation; national programs; less pollutants and nearby means of distribution; opinion leaders; financial discipline	Create professional organisations and other types of associations; elaborate a national plan with strict implementation and with reachable and clear indicators; develop awareness campaigns; create infrastructure with institutional capacity and specialists; develop hubs where people exchange ideas and sustainable development issues are presented
S40	Entire food chain as a circular process; all stakeholders responsibly understand, manage and consume food products	Education; mentality, habits; prices, exclusivity; taxing conventional producers for unsustainable food production; availability (access in terms of quantity, quality, and affordability); consumption patterns; food waste (increasing the lifecycle of food); number of producers; existence/development of sustainable food markets; legislative framework; national policies and strategies; medium–long-term thinking; misalignment of public authorities; consistency given by frequent changes in society	Change the mentalities; provide incentives for sustainable producers and tax unsustainable behaviours for all stakeholders; financial education; introduce sustainable development into the school curriculum; raise awareness and promote the benefits of sustainable food consumption
